# Colonization in North American Arid Lands: The Journey of Agarito (*Berberis trifoliolata*) Revealed by Multilocus Molecular Data and Packrat Midden Fossil Remains

**DOI:** 10.1371/journal.pone.0168933

**Published:** 2017-02-01

**Authors:** Diego F. Angulo, Leonardo D. Amarilla, Ana M. Anton, Victoria Sosa

**Affiliations:** 1 Biología Evolutiva, Instituto de Ecología AC, Xalapa, Veracruz, Mexico; 2 Instituto Multidisciplinario de Biología Vegetal, CONICET-Universidad Nacional de Córdoba, Córdoba, Argentina; National Cheng Kung University, TAIWAN

## Abstract

Here we conduct research to understand the evolutionary history of a shrubby species known as Agarito (*Berberis trifolio*lata), an endemic species to the Chihuahuan Desert. We identify genetic signatures based on plastid DNA and AFLP markers and perform niche modelling and spatial connectivity analyses as well as niche modelling based on records in packrats to elucidate whether orogenic events such as mountain range uplift in the Miocene or the contraction/expansion dynamics of vegetation in response to climate oscillations in the Pliocene/Pleistocene had an effect on evolutionary processes in Agarito. Our results of current niche modelling and palaeomodelling showed that the area currently occupied by *Berberis trifoliolata* is substantially larger than it was during the Last Interglacial period and the Last Glacial Maximum. Agarito was probably confined to small areas in the Northeastern and gradually expanded its distribution just after the Last Glacial Maximum when the weather in the Chihuahuan Desert and adjacent regions became progressively warmer and drier. The most contracted range was predicted for the Interglacial period. Populations remained in stable areas during the Last Glacial Maximum and expanded at the beginning of the Holocene. Most genetic variation occured in populations from the Sierra Madre Oriental. Two groups of haplotypes were identified: the Mexican Plateau populations and certain Northeastern populations. Haplogroups were spatially connected during the Last Glacial Maximum and separated during interglacial periods. The most important prediction of packrat middens palaeomodelling lies in the Mexican Plateau, a finding congruent with current and past niche modelling predictions for agarito and genetic results. Our results corroborate that these climate changes in the Pliocene/Pleistocene affected the evolutionary history of agarito. The journey of agarito in the Chihuahuan Desert has been dynamic, expanding and contracting its distribution range and currently occupying the largest area in its history.

## Introduction

The origin and evolution of the biota of the five North American deserts (Great Basin, Mojave, Colorado Plateau, Sonoran and Chihuahuan) have been of great interest to scientists since the last century [1], [2], [3], [4], [5], [6], [7], and their evolutionary processes have been associated with historical orogenic and climate events [8], [9], [10], [11], [12].

In the deserts of North America the uplift of mountain chains occurred in different stages from the middle of the Miocene to as recently as the Pliocene, a series of events known as the Neogene uplift (~15–2 Ma), while climate fluctuations are more recent, through the Pleistocene [1], [2], [4], [13], [6], [7], [10]. The fossil record suggests that some elements of the desert flora originated in the Tertiary in the North American deserts; however, it was not until the Quaternary that the current North American deserts were established [14], [15], [16], [17], [6], [7].

In the Mid-Miocene there was a rapid cooling along with an increased drought associated with volcanic activity, resulting in the expansion of arid and semi-arid land in North America [1], [2], [4]. The vegetation in the North American deserts shifted from subtropical humid forests to drier savannahs due to Quaternary climate oscillations [18], [19], [16], [20]. A large-scale aridification event began after the end of the Last Glacial Maximum in northern Mexico and adjacent southwestern United States, which allowed the xeric flora to establish [21], [22], [23], [13], [21], [24]. This dry xeric vegetation was abundant until 8000 years ago [25], [26], and subsequent changes to semi-desert grassland and eventually to desert shrubby vegetation have been documented [27], [21], [23], [13] [20]. The survival of species in refugia, changes in population number, size and genetic variation, and tempo and mode of recolonization during Pleistocene climate fluctuations have been well documented for the taxa of these deserts [20], [28], [29], [12], [30]. Furthermore, vicariance events related to Miocene orogenic activity (e.g. [8], [31], [9], [32]), and changes in genetic and geographic structure influenced by climate fluctuations during the Pleistocene (e.g. [33], [34], [35], [12]) have been events detected in a number of phylogeographic studies conducted on the plants and animals of the North American arid regions.

The largest and most biologically diverse desert in North America is the Chihuahuan Desert, a warm desert extending from western Texas and southern New Mexico to northern Mexico on the Mexican Plateau [36], [37], [38], [20]. Several boundaries have been proposed for Chihuahuan Desert based upon climate and vegetation and these are widely debated, mostly in the recognition of the southern limits. Some authors proposed a limit in Chihuahua [39], others in Hidalgo and Querétaro [40], [41], and still others think that areas with xeric vegetation in the Tehuacán Valley in Puebla and Oaxaca are related to the Chihuahuan Desert [42].

The Chihuahuan Desert has been considered an area rich in endemic species and centre of origin of modern desert biota [43], [6]. The climate around its southern edge is less extreme and sustains a richer flora than the northern zones [42], [14]. Two well defined geological formations are recognized within this arid region: the Mexican Plateau, a vast elevated area which has been geologically and climatically stable since the middle Miocene [43], and the Sierra Madre Oriental, a mountain range with arid vegetation at lower elevations, and with topographic complexity [44]. The Pleistocene in the Chihuahuan Desert was characterized by a cool climate [4], [16], and the Mid-Pleistocene was drier than the Late Pleistocene which was much more humid than it is at present owing to rains during winter and the presence of several large palaeolakes [19], [16]. After the Last Glacial Maximum, the climate in the Chihuahuan Desert and adjacent regions became progressively warmer and drier [18], [16]. Palaeoclimate and palaeobotanical data suggest that the arid vegetation in North America was restricted during the more humid and moderately cold pluvial periods [45], [46], allowing species to persist in certain areas of their distribution range [18], [46], [47].

To understand the processes influencing geographical patterns of genetic variation in plants of the Chihuahuan Desert, we selected the shrubby plant known as Agarito or Algerita (*Berberis trifoliolata* Moric.), a species endemic to the Chihuahuan Desert. We chose this plant because it was an important element of the chaparral of the Madro-Tertiary vegetation in northern Pacific areas from Colorado to Baja California Peninsula [48]. The fossilized remains of this species have been reported from packrat middens in western Texas and in Coahuila in the Chihuahuan Desert from the Pleistocene with precise dating that goes from about 12000 to 20000 years ago [49], [50]. Fossil leaves of *Berberis* sp. similar to *B*. *trifoliolata* have been identified from Tepexi, Puebla a southern semi-arid area peripheral to Tehuacán Valley, in central Mexico from the Oligocene [51].

Historical records, at least those from the Pleistocene, when contrasted with current records using palaeomodelling will allow us to understand whether the range of Agarito contracted or expanded. Our previous ecological niche modelling for the current distribution of this species indicated that its climate preference on the Mexican Plateau differs from its preference in the northeastern region of the Chihuahuan Desert [52].

Here, we identify genetic signatures, based on chloroplast DNA and AFLPs along with ecological niche modelling and spatial connectivity analyses using the Chihuahuan Desert shrub *Berberis trifoliolata* as a model to: (1) understand whether the extent of its distribution varied in different periods and whether these changes are linked to differences in population genetics; (2) recognize whether Miocene orogeny and/or Pleistocene/Pliocene climate fluctuations influenced the evolutionary processes in Agarito; (3) assess how historical climate change has influenced the spatial connectivity of natural populations.

## Methods

We provide here a brief overview of the methods. Extensive details are provided in S1 Methods.

### Sampling

**Fig 1:**
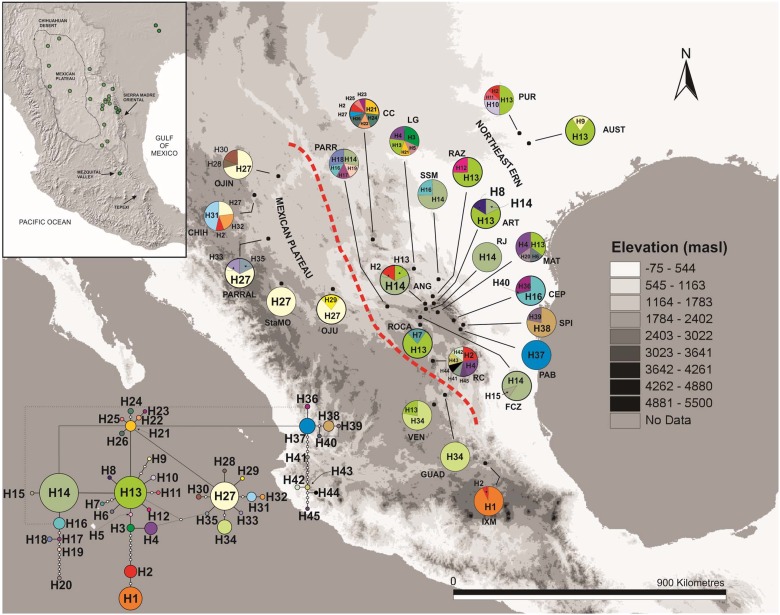
Populations of *Berberis trifoliolata* studied and their haplotypes. Pie charts represent the haplotypes found in each sampling locality. Size of sections is proportional to the number of individuals with that haplotype. The size of circles in the haplotype network is proportional to the frequency of each haplotype. Population names are given in Table 1. Red line shows the division between the Mexican Plateau and the Northeastern populations.

We obtained collecting permits to conduct this work from the Secretaría de Medio Ambiente y Recursos Naturales, Instituto Nacional de Ecología, Dirección de Vida Silvestre (permit number: Registro de Colección Científica VER-FLO-228-09-09. A total of 208 individuals of *Berberis trifoliolata* were sampled from 25 localities that we considered populations comprising its current distribution range (Fig 1, Table 1). All relevant data are within the manuscript, its supporting information files, and GenBank (accessions numbers can be found in S1 Table.

**Table 1:** Sampling information of the populations of *Berberis trifoliolata* studied: country and locality were provided followed by population abbreviation, number of individuals for DNA sequences, number of individuals for AFLPs, latitude and longitude and their respective haplotypes.

Sample location (Abbreviation)	*N*_ind_*Cp*	*N*_*ind*_*AFLPs*	N. Latitude	W. Longitude	Haplotype
U.S. Austin, Texas (AUST)	7	5	30.142833	-97.96271	H9, H13
U. S. Purola, Texas (PUR)	8	5	30.48536	-98.28256	H2, H10, H11, H13
Mexico, borderline Coah-Zac (FCZ)	13	5	24.98063	-101.1795278	H14, H15
Mexico, Rancho Jaguey, Coah (RJ)	12	5	25.23051	-101.019309	H14
Mexico, Parras, Coah (PARR)	7	5	25.36014	-102.17508	H14, H16, H17, H18, H19
Mexico, Arteaga, Coah (ART)	4	5	25.39958	-100.79894	H8, H13, H14
México, La Angostura, Coah (ANG)	7	6	25.33953	-101.04506	H2, H13, H14
México, Ramos Arizpe, Coah (RAZ)	4	4	25.61408	-100.83078	H12, H13
Mexico, La Gavia, Coah (LG)	9	-	26.34683	-101.36172	H3, H4, H5, H13, H21
Mexico, Cuatro Cienegas, Coah (CC)	15	5	27.30289	-102.61372	H2, H21, H22, H23, H24, H25, H26, H27
Mexico, Sierra de San Miguel, NL (SSM)	5	5	26.11144	-100.65544	H14, H16
Mexico, Near Matehuala, NL (MAT)	8	-	25.13863	-100.68811	H4, H6, H13, H20
Mexico, Pablillo, NL (PAB)	12	6	24.60978	-100.00183	H37
Mexico, Cerro El Potosí, NL (CEP)	7	10	24.88603	-100.18894	H16, H36, H40
Mexico, Rocamontes, Dgo (ROCA)	5	5	24.74164	-101.17501	H7, H13
Mexico, San Pedro Iturbide, NL (SPI)	6	6	24.724	-99.90897	H38, H39
Mexico, Parral, Chih (PARRAL)	6	5	27.32045	-105.71926	H27, H33, H35
Mexico, Ojinaga, Chih (OJIN)	11	5	29.1491	-105.39052	H27, H28, H30
Mexico, Near Chihuahua city (CHIH)	9	-	28.59815	-106.11728	H2, H27, H31, H32
Mexico, Santa María del Oro, Dgo (StaMO)	5	4	25.98615	-105.32809	H27
Mexico, Ojuelas, Dgo (OJU)	5	4	25.79861	-103.78402	H27, H29
Mexico, Ixmiquilpan, Hgo (IXM)	18	6	20.61348	-99.23509	H1, H2
Mexico, Guadalcazar, SLP (GUAD)	2	-	22.65183	-100.43486	H34
Mexico, Ventura, SLP (VEN)	10	5	22.38197	-100.77363	H13, H34
Mexico, Real de Catorce, SLP (RC)	13	5	23.73769	-100.8455	H2, H4, H41, H42, H43, H44, H45

San Luis Potosí (SLP), Coah (Coahuila), Zacatecas (Zac), Nuevo Leon (NL), Durango (Dgo), Chihuahua (Chih), Hidalgo (Hgo).

### Phylogenetic reconstruction and population genetic analyses

Phylogenetic relationships among chorloplast (cp) DNA haplotypes were reconstructed using Bayesian Inference (BI) using MrBayes 3.1.2 [53]. To examine intraspecific relationships between sampling localities, unrooted haplotype networks were built with TCS v.1.21 [54] based on a 95% parsimony criterion. A spatial molecular variance analysis was performed using SAMOVA 1.0 [55], which determines groups of populations that are geographically homogeneous. The number of groups ranged from 2 to 5, and the values of fixation indices were compared among different group numbers with 1,000 permutations.

Parameters of population diversity, including haplotype diversity (h) [56], and nucleotide diversity (*π*) [56] were calculated using DnaSP v. 5.0 [57]. To investigate the hierarchical levels of the population structure, *F*_*ST*_ values were obtained from an analysis of molecular variance (AMOVA) run in Arlequin [58]. Sampling localities were grouped as a) a single population b) groups identified by SAMOVA analysis and c) groups accord to geographical area as Mexican Plateau vs Northeastern (including the Sierra Madre Oriental and adjacent areas), to determine the amount of variation among and within these regions. Significance levels were calculated with 10000 nonparametric permutations.

### Divergence time

The time of divergence for intraspecific diversification of *Berberis trifoliolata* was estimated using a Bayesian approach implemented in BEAST v.1.6.1 [59]. Dataset included the same outgroup used on Bayesian analysis. Calibration of the genetic tree was performed using the substitution rate of plastid DNA. The cpDNA substitution rate of most angiosperm has been estimated to vary between 1 and 3x10^-9^ substitutions/site/year [60]. Because of the uncertainty of the rates, we used an evolutionary rate of 2.0x10^-9^ substitutions/site/year to estimate divergence time assuming a coalescent model with expansion population growth.

### Demographic and spatial analyses

Tajima’s *D* [61], Fu’s *Fs* [62] and *R*_*2*_ [63], are among the most powerful selective neutrality tests when there is no recombination [64], [63] and were calculated to detect past demographic range expansion events using Arlequin. Then, a mismatch distribution of pairwise nucleotide differences [65], was calculated and compared with expected values for an expanding population using the sum of squared deviations (SSD) [66]. Mismatch analysis was performed in DnaSP to distinguish between models invoking past exponential growth versus historical population stasis. To understand past population dynamics, such as changes in relative effective population size over time, a Bayesian skyline plot analysis was carried out using BEAST 1.6.1 [59].

We evaluated the spatial genetic structure in populations of *Berberis trifoliolata* by interpolating pairwise genetic distances across the landscape. We calculated genetic distances among sampling sites using the Delaunay triangulation-based connectivity network in the program Alleles In Space [67]. The genetic distances were imported into ArcView GIS 3.2 (Environmental Systems Research Institute Inc. 1999. Redlands, California) and the interpolation was carried out using the Spline method with default parameters.

### AFLP analyses

AFLP markers were amplified following Vos *et al*. [68]. Selective amplifications were performed using the primer combinations of Roy *et al*. [69].

We examined the patterns of population structure in two ways. First, the assignment probability test in Structure v.2.2 [70], was carried out to reveal hierarchical sub-structuring running separate subsequent analyses for each group previously identified. Then the second analysis was a Principal Coordinate Analysis (PCoA) using the genetic distance matrix generated from the binary presence-absence matrix of the AFLPs as implemented in GenAlex. We performed analyses of Molecular Variance (AMOVA) both within the two biogeographical provinces (Mexican Plateau vs Northeastern) and using the groups identified by Structure and PCoA.

### Ecological niche modelling

The ecological niche was estimated based on distribution. Nineteen environmental variables derived from temperature and precipitation data were obtained from WorldClim 1.4 [71] at a resolution of 1 km^2^. A correlation analysis was performed to eliminate correlated environmental variables using the program PAST v.2.12 [72] and only the eleven least correlated variables (Pearson ≤ 0.7 based on all sample locations, see [73]) were used (S2 Table). A total of 91 records were compiled during the fieldwork of this project, and these were added to specimens from the following herbaria: IEB, ANSM, ENCB, MEXU, XAL. Additional records were compiled virtually from TEX, LL and ARIZ Herbaria. Records separated by less than a kilometre from an already included location or with no georeference data were excluded. To identify potential areas where the species would have survived during the Last Interglacial (140,000–120,000 years ago) and during the extreme conditions of the Last Glacial Maximum (LGM) (about 22,000 years ago), the present ecological niche of *B*. *trifoliolata* was modelled and projected onto these past periods using MaxEnt v. 3.3.2 [74]. For the LGM analysis we used general circulation model simulations from two coupled climate models that have been used in previous studies (e.g., [75], [76], [77], [78]: the Community Climate System Model (CCSM) [79] and the Model for Interdisciplinary Research on Climate (MIROC) [80].

In addition, the past ecological niche based on the fossil records of packrat middens was modelled to corroborate the Last Glacial Maximum CCSM and MIROC models for *Berberis trifoliolata*. We only used packrat middens dating to the LGM (22,000–14,000 years ago, S3 Table). To test the predictability of ecological niche and distribution among time periods, niche models were constructed for the Pleistocene using CCSM and MIROC climatic models (see above) and projected onto present-day climate conditions using MaxEnt v. 3.3.2 [74].

### Spatial connectivity

We used Circuitscape 3.1 to calculate a resistance distance statistic that summarizes overall connectivity between each pair of populations [81]. Circuitscape considers the landscape to be an electrical circuit, in which populations serve as sources or sinks for electrical current, while landscape features either inhibit or assist the flow of that current by offering a high or low resistance to the circuit(s) connecting the populations [82], [83], [81]. Each Species Distribution Modelling (SDM) raster (Current, CCSM and MIROC) was imported to into Circuitscape, and a conductance grid in which higher cell values denote greater ease of movement was chosen, and a connection scheme that allowed gene flow among the nearest cells was applied.

### Rarefaction analysis

To determine whether sample sizes adequately represented population genetic variability, rarefaction curves were generated to qualitatively assess the proportion of haplotypic richness sampled at each group recovered. Computations were performed in EstimateS v. 9.0 [84] using 1,000 replications.

A trend towards an asymptotic relationship infers haplotype saturation. In contrast, a steep slope suggests that a large fraction of the available haplotype diversity remains unsampled.

## Results

### Phylogenetic reconstruction and haplotype network

The length of the two combined plastid markers (*trnL-rpl32* and *psbA–trnH*) was 1,156 bp. An unreliable hypervariable SSR (position 233–258) was removed from the matrix. Reliable identified gaps were recoded and treated as a single mutation. Forty-five haplotypes were identified in the statistical parsimony network (Fig 1, Table 1). Some haplotypes were common and widespread like H13 and H14, more commonly in the Northeastern populations. On the Mexican Plateau only haplotype H27 was widespread. Haplogroups were connected by one step in the H27 group, while in H14-H13 group they were connected by several steps (Fig 1). Of the Northeastern populations, CC had the highest number of haplotypes in the same H13-H14 haplogroup, including as well the H27 haplogroup, while the CHIH population had the highest number of haplotypes for the Mexican Plateau (Fig 1, Table 1). In Northeastern populations 11.7% were private haplotypes (e.g. the haplotypes H36 and H40 were private for CEP, while haplotypes H38 and H39 were private for SPI). Only 8.7% were private haplotypes for the Mexican Plateau (e.g. haplotype H1 was found only in IXM, the Agarito southernmost population, while haplotypes H33 and H35 were private to PARRAL; Fig 1). Haplotype H2 was frequently found in distant populations for both the Northeastern and also the Mexican Plateau (e.g. IXM and CC) (Fig 1).

The 50% majority consensus tree (results not shown) grouped all *Berberis trifoliolata* haplotypes in a single, well supported clade (PP = 1). The relationship within *B*. *trifoliolata* haplotypes was not resolved and only one haplotype group with a modest support (PP = 90) that included north-eastern populations (haplotypes H20, H41 to H45) and Mexican Plateau populations (haplotypes H1 and H2) was recovered. No further haplotype groups were supported by the BI analysis.

SAMOVA results revealed significant *F*_*CT*_ values for groups between K = 2 and K = 5, with the highest *F*_*CT*_ values for K = 3 (Table 2). The three groups identified correspond to population RC (within north-easthern geographic group), IXM (within Mexican Plateau geographic group) and a third group was formed by all other populations (mixed populations of both geographical regions) suggesting one large population. An additional increase in K number led to a smaller *F*_*CT*_ value and dissolution of group structure.

**Table 2:** Spatial analysis of molecular variance (SAMOVA) for *Berberis trifoliolata* populations.

Groups tested	Percentage of variation			Fixation indices		
	Among groups	Among populations within groups	Within populations	FSC	FST	FCT
II	72.98	20.35	6.66	0.75**	0.93**	0.73*
III	76.65	16.23	7.12	0.69**	0.93**	0.77**
IV	75.6	16.86	7.53	0.69**	0.92**	0.75**
V	74.25	17.38	8.36	0.67**	0.91**	0.74**

*P<0.05,

**<0.0001

### Divergence time

**Fig 2:**
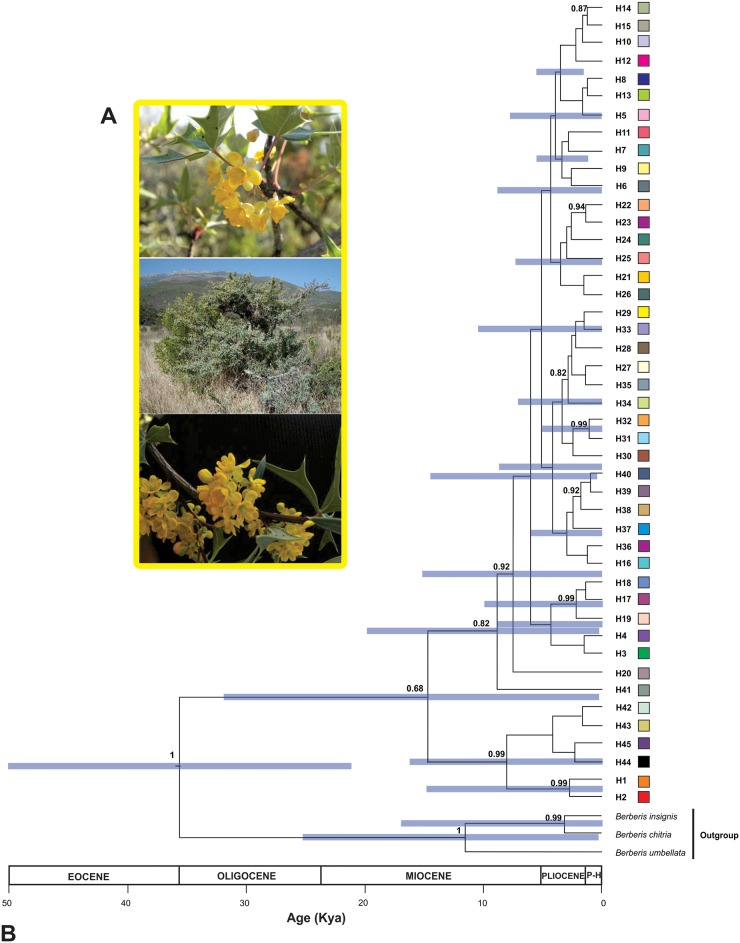
A) Flowers and habit of *Berberis trifoliolata*. B) Bayesian chronogram showing the time of divergence Agarito haplotypes. Colours of the squares correspond to those of Fig 1. The 95% credibility intervals are shown by purple bars.

Estimation of time of divergence for *Berberis trifoliolata*’s populations shows that the basal split between clade containing haplotypes of Ixmiquilpan (IXM: H1, H2) and Real de Catorce (RC: H42, H43, H44, H45) vs. remaining haplotypes occurred in the late Pleistocene at ca. 15 kya (95% HPD, 31.9–0.02 kya). The major intra-specific clades diverged during early Holocene (c. 10 kya to present). Then, the main divergences within North and remaining haplotypes of the Chihuahuan desert including Mexican Plateau haplotypes have occurred at *ca*. 9–6 kya. Later the haplotypes find in the Eastern of Sierra Madre Oriental diverged from Mexican Plateau haplotypes *ca*. 4 kya (95% HPD, 10.6–0.01 kya). Divergence estimates for *Berberis trifoliolata* populations using a coalescent model and assuming expansion population growth are shown in Fig 2.

### Genetic analyses

Parameters of population diversity, including haplotype diversity (h), and nucleotide diversity (*π*) were calculated for the two geographical groups selected a priori within the Chihuahuan Desert (Mexican Plateau and Northeastern region). Nucleotide diversity values were very low in both groups of populations (*π* = 0.003 and *π* = 0.005 for Northeastern and Mexican Plateau, respectively) (Table 3). The highest haplotype diversity was found within Northeastern populations (*h* = 0.86), in contrast to the Mexican Plateau populations with lower diversity (*h* = 0.79).

**Table 3:** Results of genetic and demographic analyses of *Berberis trifoliolata*'s phylogroups. Haplotype diversity (h), nucleotide diversity (π), Fu’s FS (Fs), Tajima’s D (DT) and Ramos-Onsins and Rozas (R_2_) are indicated.

Ecoregion	Parameters	*rpl32-trnL*^UAG^	*trnH-psbA*	Combined
**Northeastern**	*h* (± SD)	0.78 (± 0.02)	0.74 (± 0.03)	0.86 (± 0.02)
	*π* (± SD)	0.0038 (± 0.63E-3)	0.0027 (± 0.26E-3)	0.003 (± 0.4E-3)
	Fs	-0.99	-3.66**	-3.13**
	D_T_	-1.38*	-1.91**	-1.87**
	R_2_	0.04*	0.03**	0.03**
**Mexican Plateau**	*h* (± SD)	0.64 (± 0.05)	0.78 (± 0.03)	0.79 (± 0.04)
	*π* (± SD)	0.0065 (± 0.9E-3)	0.0032 (± 0.42E-3)	0.005 (± 0.54E-3)
	Fs	1.82	-2.14	0.82
	D_T_	1.59	-1.24	0.85
	R_2_	0.16	0.06	0.13

* P<0.01

** P<0.001

The AMOVA results when *Berberis trifoliolata* populations were treated as a single group indicates that 82.64% (P<0.0001) of the variation can be explained by differences among populations (Table 4). Strong population structure with highest *F*_*CT*_ value was obtained when samples were grouped by SAMOVA results (*F*_*CT*_ = 0.76). However, when samples were grouped as separated by the geographical regions a significant but smaller proportion of the variation can be explained by differences among populations within groups (*F*_*CT*_ = 0.27; Table 4).

**Table 4:** Analysis of molecular variance (AMOVAs) performed among groups of *Berberis trifoliolata* populations.

Source of variation	Percentage of variation	*F*-statistic
***CpDNA***		
**No group defined**		
among populations	82.64	FST = 0.82**
within populations	17.36	
**SAMOVA**		
among groups	76.65	FCT = 0.76**
among populations within groups	16.23	FSC = 0.69**
within populations	7.12	FST = 0.92**
**Northeastern vs. Mexican Plateau**		
among groups	27.32	FCT = 0.27**
among populations within groups	57.86	FSC = 0.79**
within populations	14.82	FST = 0.85**
***AFLPs***		
**No group defined**		
among populations	44.98	FST = 0.45**
within populations	55.02	
**Groups according Structure**		
among groups	24.06	FCT = 0.24**
among populations within groups	23.77	FSC = 0.31**
within populations	52.17	FST = 0.47**
**Northeastern vs. Mexican Plateau**		
among groups	4.74	FCT = 0.04
among populations within groups	23.69	FSC = 0.44**
within populations	29.91	FST = 0.47**

d.f., degrees of freedom; FCT, differentiation among groups within the species; FSC, differentiation among populations within groups; FST, differentiation among populations within the species.

**P < 0.0001

### Demographic and spatial analyses

To test explicit a priori hypotheses of genetic structure and demographic change by regions [52], demographic analyses were carried out grouping populations by geographical regions within Chihuahuan desert (Mexican Plateau/Northeastern population). Groups suggested by SAMOVA were not considered for demographic analysis because two groups have a single populations with few individuals sampled.

The results for Tajima’s D, Fu’s F and Ramos-Onsins and Rozas’s *R*_*2*_ tests indicate that the populations deviate from the null model of a constant population size (Table 3), providing evidence for range expansion in the Northeastern populations.

**Fig 3:**
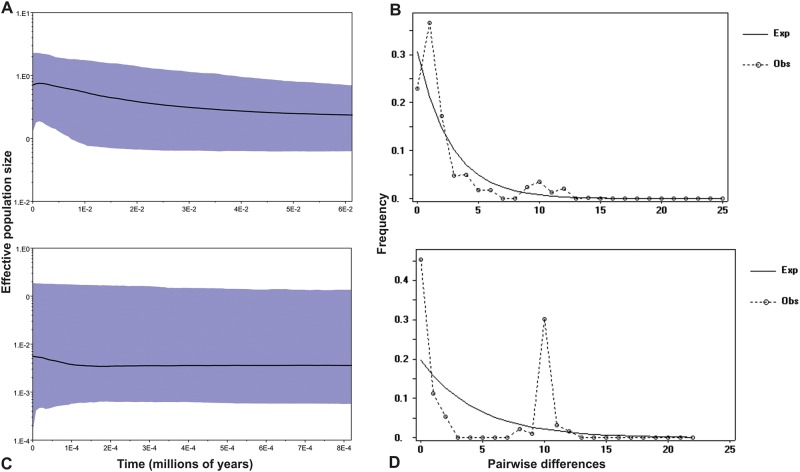
Results of the Bayesian skyline plots (left) and Mismatch distribution (right). A and B correspond to Northeastern populations and C and D corresponds to Mexican Plateau populations. Solid lines in A and C are estimates of means.

Bayesian skyline plots indicate that the population size of Northeastern group increased slightly, whereas that of the Mexican Plateau group was nearly constant (Fig 3A). The mismatch distribution analyses of Northeastern populations produced a distinctive unimodal curve, providing evidence that the populations underwent a bottleneck followed by a sudden demographic expansion (P_ssd_>0.05) (Fig 3B). Neither the Neutrality nor the mismatch distribution analyses (Fig 3C and 3D) provided evidence of past population growth for the Mexican Plateau populations.

The interpolated pairwise genetic distances shown in Fig 3 revealed strong genetic differentiation among Northeastern and Mexican Plateau populations (S1 Fig). The black shading indicates a high degree of genetic divergence, as occurs in CC, LG and PARR populations in the Northeastern group and in OJIN, PARRAL and OJU populations on the Mexican Plateau, suggesting that they have remained stable for long periods of time. In the southern populations of the Northeastern group, grey shading indicates a low degree of genetic differentiation, which is consistent with recent range expansion (S1 Fig).

### AFLP analyses

The reproducibility test was carried out with primer combinations *Mse*I-CTG/*Eco*RI-ACA and *Mse*I-CTC/*Eco*RI-ACA using two randomly selected DNA samples. A high degree of reproducibility (reproducibility score = 98%) was obtained for each of the primer combinations tested (data not shown). All markers shared between populations were non-redundant and had frequencies exceeding 5%.

**Fig 4:**
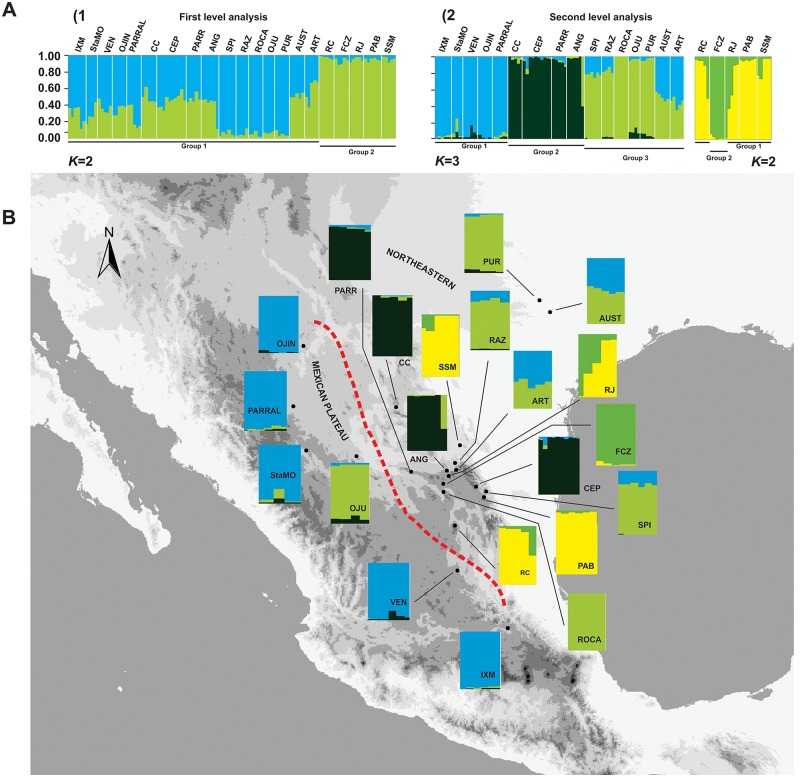
Bayesian assignment analysis showing the spatial genetic structure of *Berberis trifoliolata* populations. A) Bar plots depicting the results of two rounds of hierarchical STRUCTURE analyses A1) First level of analysis showing *K* = 2 fit the genetic groups the best A2) Second level of analysis showing substructure. White vertical lines separate sets of individuals of different populations. B) Map showing the populations sampled and the proportion of the genome as determined by second-level STRUCTURE analysis. The codes indicate sampling localities given in Table 1.

The spatial genetic structure of *Berberis trifoliolata* populations shown by the Bayesian assignment based on *ΔK* values revealed a clear population substructure across hierarchical levels (Fig 4A-1 and 4A-2). The first round of analyses revealed that *K* = 2 fit the genetic groups the best (first level; Fig 4A-1). However, only a group belonging to Northeastern populations (RC, FCZ, RJ, PAB, SSM; green cluster) was preserved in each independent run. The rest of populations of the Northeastern group and populations of the Mexican Plateau were genetically intermixed and no clear patterns were found along independent runs (first level; Fig 4A-1). A second round of analyses was performed using the results retrieved by previous analyses and later re-analyzed separately. First we selected the cluster with greater allocation (light green group) because this group remained stable for each *K* value analyzed. Then we took into consideration the other populations that were genetically intermixed and unstable in independent runs (see Fig 4A-1). The second round shows that two groups were recognized for the first light green group, whereas the remaining populations of the first result were assigned to three main clusters (second level; Fig 4A-2). At *K* = 3 groups, the first group contained the Mexican Plateau (light blue cluster). Some Northeastern populations (CC, CEP, PARR and ANG) formed a second group (dark green cluster), while the SPI, RAZ, ROCA, OJU, PUR, AUST and ART populations in the Northeastern group were assigned to the third group (light green cluster), but slightly mixed with Mexican Plateau genotypes (light blue). The *K* = 2 groups were formed by RC, RJ, PAB and SSM (yellow cluster), but slightly mixed with the strong green genotype, whereas FCZ was formed mainly by the strong green genotype (second level; Fig 4A-2).

The PCoA analysis shows the variation in genetic distances among populations and identified four clusters partially congruent with results of the Structure analyses (S2 Fig); the groups in this case having some geographic correspondence. The PCoA graph shows the Northeastern populations grouped mainly in the lower quadrants, while the Mexican Plateau populations are in the upper right quadrants. The Northeastern populations are grouped into four clusters (S2 Fig).

In AMOVA analyses, when all population were considered as a single group the variation was explained by differences within populations (55%). The *F*_*CT*_ value of 0.24 (P<0.001) indicates a significant but smaller proportion of the variation when the samples were grouped by the Structure results (Table 4). However, when populations were grouped by geographical regions a weak but significant genetic differentiation can be detected within populations (*F*_*CT*_ = 0.04; Table 4).

### Niche based distribution modelling

**Fig 5:**
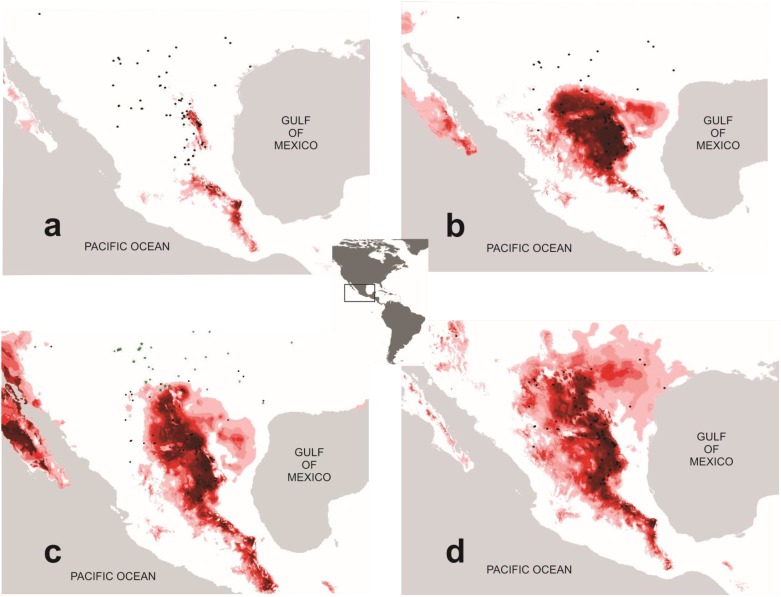
Ecological niche modelling for *Berberis trifoliolata*. Black dots indicate sampled localities. A) Current potential distribution. B) Predicted distribution during the Interglacial period. C) Predicted distribution during the Last Glacial Maximum (CCSM model). D) Predicted distribution during the Last Glacial Maximum (MIROC model). Blue dots indicate records without coordinates. Green stars indicate packrats middens containing Quaternary fossil remains of agarito. Color range from pink to light red to red colors indicates low, medium and high probability, respectively.

The AUC value was 0.90 to 0.94, indicating good performance by the model. The potential distribution of *B*. *trifoliolata* during the Last Interglacial period was fragmented, with small areas of suitable habitat in the folds of the Sierra Madre Oriental, and in the north, in Nuevo León and Coahuila (Fig 5A). A second potential area that was predicted is the Mezquital Valley in Hidalgo. The Tehuacán Valley is predicted as well for the Last Interglacial period projection. The Last Glacial Maximum projection placed suitable habitats further south in the central part of the Sierra Madre Oriental (Fig 5B and 5C). There was a greater difference between the Current and Last Glacial Maximum niche models for the CCSM model than for the MIROC model (Fig 5).

Niche modelling for current climate conditions predicted most of the current geographic distribution of *Berberis trifoliolata* (Fig 5D). However, it over-predicted the distribution in the Tehuacán Valley region where this species is not currently found. The predicted probability of occurrence for eastern Texas, Arizona and the Mexican Plateau was low.

Niche models for the packrat middens with records of agarito were highly accurate when CCSM layers were projected, reflecting its current distribution (S3A Fig). In contrast, using MIROC layers, models predicted that agarito probably occupied a smaller area in the Chihuahuan Desert and over-predicted its distribution in North America (S3B Fig). Predictions were similar for agarito using the Last Glacial Maximum layers, with the CCSM layers over-predicting its distribution in North America and in the Sonoran Desert (S3C Fig), and MIROC over-predicting the Pacific coast in Mexico and some areas in North America (S3D Fig). The CCSM and MIROC layers in the Last Glacial Maximum both predict the distribution of agarito on the Mexican Plateau (S3C and S3D Fig).

### Spatial connectivity

**Fig 6:**
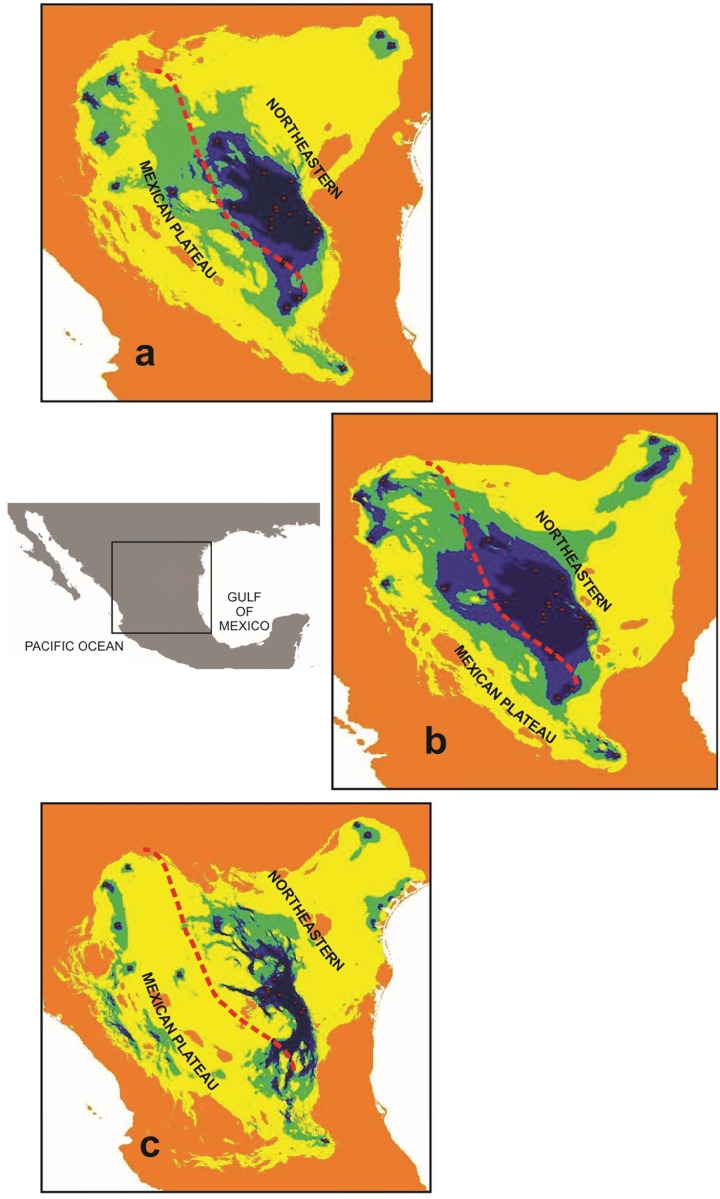
Connectivity maps among populations for *Berberis trifoliolata*. Colder (blue) colours indicate areas with stronger connectivity; areas where connectivity is tenuous are shown in warmer colours. A) Present; B) Last Glacial Maximum (CCSM model); C) Last Interglacial. The red line delimits the division between the Mexican Plateau and Northeastern groups of populations.

Our results for the climatic spatial resistance surfaces suggest that connectivity among populations in the Northeastern group was strong (in blue colour) in all three analyses: Current, Last Glacial Maximum and Last Interglacial (Fig 6A, 6B and 6C). Low connectivity was detected among Mexican Plateau populations in the three analyses: Current, Last Glacial Maximum and Last Interglacial (Fig 6A, 6B and 6C). Connectivity was greatest between the Northeastern and Mexican Plateau groups during the Last Glacial Maximum (Fig 6B). Texas and the southernmost populations remained isolated and were not connected to the other populations in the Current, Last Glacial Maximum or Last Interglacial period (Fig 6A). The lowest degree of connectivity within Northeastern populations and also within the Mexican Plateau populations was detected during the Last Interglacial period (Fig 6C). Our results for this period suggest as well that connectivity was restricted to nearby populations. The Northeastern and northern Mexican Plateau populations were completely disconnected in during the Last Interglacial (Fig 6C).

### Rarefaction analysis

Rarefaction curves when all populations were considered provided evidence that haplotype richness has reached an asymptote, which could be suggesting haplotype saturation. However the two haplotype rarefaction curves representing each geographic region (Mexican Plateau and Northeastern) provided strong evidence that more haplotypes would be revealed by increasing the number of individuals sequenced (see S4 Fig).

## Discussion

### Phylogeography

Our AFLP results showed a clear change of genotypes in close Northeastern populations and between Mexican Plateau and certain Northeastern populations. This is congruent with our previous ecological niche models that identified the same two groups as having different climate preferences [52]. It has been suggested that ecological niche plays a role in processes such as speciation, indicating that niche shifts might subsequently promote parapatric speciation [85], [86]. This is likely the case for *Berberis trifoliolata*, in which incipient speciation has been detected involving the two groups of populations.

Although our AFLP results showed a genetic break between the Mexican Plateau and Northeastern populations, it was not identified by our cpDNA markers. This pattern of inconsistency has been found in other plants such like *Edraianthus graminifolius* (Campanulaceae) growing in Mediterranean mountains and it might be related to the inheritance of these markers, uniparental in the chloroplast markers [87]. Furthermore differences in genetic structure revealed by the AFLP and plastid DNA of populations has been often reported in several plants and attributed mostly to gene flow by pollination (e.g. [88], [89], [90]). Nevertheless, AFLPs are fast-evolving markers, probably suggesting a more recent divergence compared with plastid sequences [91]. Despite that these markers can not be used in Bayesian dating analyses, and based on previous studies we suggest that their signal probably reflects a Late Quaternary differentiation possibly as recent as the Last Glacial Maximum [78], while differentiation pattern found in the cpDNA data was earlier within the Pliocene-Pleistocene. In summary, we suggest that the inconsistency between the AFLP and cpDNA marker signals in agarito might be the result of two population processes, historical variation in pollen-mediated gene flow (nuclear AFLPs with Mendelian inheritance), and in long distance seed dispersal (cpDNA with maternal inheritance) with signal at different time scales (cpDNA reflecting Pliocene-Pleistocene events and AFLPs reflecting events during Last Glacial Maximum).

With regard to results on Agarito genetic structure discovered by our analyses we suggest two hypotheses. A lack of geographic structure and elevated haplotype diversity within cpDNA markers might be related to long-term persistence of Agarito populations in certain areas implying incomplete lineage sorting during population divergence events. An alternative explanation is a strong genetic flow betwen Agarito populations throughout their distribution range due to the lack of geographic barriers behaving like a panmictic population. This strong genetic flow might be related to pollination of *Berberis trifoliolata* that is pollinated by bees (most populations by the invasive *Apis mellifera*), with showy and fleshy fruit dispersed by birds [92], [93].

The highest level of genetic diversity within agarito populations shown by presence of private haplotypes as well as presence of the most widespread haplotype occurred in the Northeastern group. A set of populations within this group is located at middle elevations, from approximately 1,700 to 2,400 m a.s.l. in the Sierra Madre Oriental, a mountain chain that is complex in both its geology and its climate. Some of these populations have an elevated number of haplotypes others share rare haplotypes, like the most southern and the most northern populations. This diversity and these shared rare haplotypes might be indicative of areas that acted as refuges during the cool climate of the Pleistocene and where populations remained during interglacial periods before expanding again. Furthermore some areas in this Sierra acted as refugia for other plants [47], [94], [78]. Therefore we suggest that geographic patterns identified in Agarito are probably the response to multiple factors acting on either demography or population genetic structure. Post-glacial recolonization and more recent shifts induced by regional climate change could be playing a major role in the structuring of geographical genetic patterns.

In contrast, the Mexican Plateau populations have lower levels of genetic diversity but higher levels of genetic differentiation among populations, and show no evidence of population expansion. These patterns might be reflecting a founder effect or else are consistent with population stability through time, allowing for the accumulation of mutations and genetic divergence among adjacent populations (e.g. [95], [28], [96]. The climate of the Mexican Plateau, has been stable since the Middle Miocene [43].

### Current and palaeodistribution modelling, and packrat midden predictions

Our results of current niche modelling and palaeomodelling showed that the area currently occupied by *Berberis trifoliolata* is substantially larger than it was during the Last Interglacial period and the Last Glacial Maximum, suggesting that it has been gradually expanding northward since the Last Interglacial period. These results broadly agree with the results of our genetic analyses.

Our palaeoclimatic projections for the Last Glacial Maximum did not predict areas of distribution of agarito in the northern Chihuahuan Desert, southern Texas, or on the Mexican Plateau. As concluded in palaeoclimate and palaeobotanical studies, during the more humid and moderately cold pluvial periods of the Last Glacial Maximum in North America arid vegetation was replaced by an assemblage of plants currently found at higher elevations or was restricted to portions of their distribution range [45], [46]. Our results suggest that agarito remained confined to Northeastern areas and expanded its distribution just after the Last Glacial Maximum when the weather in the Chihuahuan Desert and adjacent regions became progressively warmer and drier [18], [16]. This hypothesis is consistent with our results of molecular dating and Bayesian Skyland Plots that suggest a cladogenesis event and an increment of population size respectively after the Last Glacial Maximum, at the beginning of the Holocene.

Weak predictions for the current distribution of agarito in Texas and in the Mexican Plateau may also suggest that the populations are expanding to new areas. The density of *B*. *trifoliata'*s populations in Texas rangelands has increased during the last century owing to fire suppression, overgrazing, and climate change [97], [98], [99]. In addition, the most extensive over-prediction by the Current and palaeoclimatic modelling in the south occurred for the Tehuacán Valley, where live specimens of Agarito have never been recorded in modern times. As mentioned above, fossil leaves of *Berberis* sp. similar to *B*. *trifoliolata* were reported from the Oligocene in Tepexi, Puebla, an area bordering the west of the Tehuacán Valley suggesting that the ancestors of *B*. *trifoliolata* might have had an extended range of distribution in this region.

Niche predictions based on packrat middens helped us elucidate whether the fossil remains of *Berberis trifoliolata* found in the *Neotoma* nests predict its distribution in current and Interglacial periods. Packrat middens fossil remains with agarito have been found only in its northern area of distribution, in the northern Chihuahuan Desert and in some other areas in Texas, and the models over-predicted northern areas in North America. However the most important prediction is for the Mexican Plateau and is congruent with current and past niche modelling predictions for agarito and genetic results.

### Spatial connectivity and the journey of agarito in North America

Climatic spatial resistance surface analyses for the Current, the Last Glacial Maximum and Last Interglacial period indicate that there were corridors between neighbouring agarito populations in the Northeastern group, but there was no connectivity among the Mexican Plateau populations, and only a low degree of connectivity is indicated for the past. Another gap in connectivity was identified between the southernmost populations with the populations of the rest of the Mexican Plateau in all three palaeomodels. The greatest connectivity seems to have occurred during the Last Glacial Maximum, when the climate was colder with prolonged lower temperatures and precipitation [100], [101], [102], [12]. These results could suggest that agarito was able to survive glacial events, probably reducing its distribution in the north and connecting (greater gene flow) the Northeastern populations with those of the Mexican Plateau. These results agree with those of previously published studies (e.g. [103], [104]).

Our niche modelling suggests that the current distribution of *Berberis trifoliolata* is the most extensive it has ever been, occupying areas in Texas, the Chihuahuan Desert, the Sierra Madre Oriental mountain range, the Mexican Plateau and its southernmost area, the Mezquital. It also grows on the eastern slopes of the Sierra Madre Oriental in semi-arid vegetation outside the limits of the Chihuahuan Desert (see Fig 1). The occurrence of *Berberis trifoliolata* fossil remains in packrat middens during the Last Glacial Maximum (22,000–14,000) indicates that this shrubby plant inhabited arid habitats in the northern Chihuahuan Desert and additional areas in Texas. Our phylogeographic, genetic, demographic, spatial analyses and our ecological niche modelling for agarito and packrat middens suggest that agarito was able to survive glacial events by reducing its range of distribution in the north and populations in the Mexican Plateau were connected with these northern populations. Moreover, rare and diverse haplotypes in populations of the Northeastern group might indicate that they remained in certain areas. The most noteworthy expansion was northward from the time of the Last Interglacial period. Agarito populations were spatially separated during the interglacial periods and later reconnected during the Last Glacial Maximum. It has been suggested that Pleistocene climate fluctuations promoted range expansion in plants from the warm deserts of North America, like the Chihuahuan Desert [105], [30]. Our results corroborate that these climate changes in the Pliocene/Pleistocene had an effect on the evolutionary history of agarito. The journey of agarito in the Chihuahuan Desert has been dynamic, expanding and contracting its distribution range and currently occupying the largest area in its history.

## Supporting Information

S1 Dataset Molecular data matrix used in analyses. (FAS)

S1 Methods Detailed methods used in this study. (DOCX)

S1 Table List of GenBank accession for cpDNA haplotypes. (DOCX)

S2 Table Uncorrelated environmental variables used in the current and palaeodistribution modelling in *Berberis trifoliolata*. (DOCX)

S3 Table Samples used for Packrat Middens models prediction. (DOCX)

S1 Fig Interpolated pairwise genetic distances within *Berberis trifoliolata* populations using a 30 sec (c. 1 km) grid size. Shading is proportional to genetic distance, with short genetic distances in white and greater distances in black. The white line shows division between the Mexican Plateau and Northeastern populations.
(TIF)

S2 Fig PCoA graph of AFLP genetic analyses of populations of *Berberis trifoliolata*. (TIF)

S3 Fig Summary of model predictions based on Last Glacial Maximum (LGM) occurrence data and climate information. A) LGM projected onto current climate conditions using the CCSM model. B) LGM projected onto current climate conditions using the MIROC model. C) Predicted distribution during the LGM using the CCSM model. D) Predicted distribution during LGM using the MIROC model. Green dots are known occurrence points for *Berberis trifoliolata* fossils found inside packrats middens from the LGM. Range of color from pink to light red to red indicates low, medium, and high probability, respectively.
(TIF)

S4 Fig Rarefaction analyses of cpDNA haplotypes based on randon sampling of *Berberis trifoliolata* dataset. (TIF)
