# Supplementary material for: Colonization in North American Arid Lands: The Journey of Agarito (Berberis trifoliolata) Revealed by Multilocus Molecular Data and Packrat Midden Fossil Remains
Source: PLoS One. 2017 Feb 1;12(2):e0168933. doi: 10.1371/journal.pone.0168933 (PMC5287450; doi:10.1371/journal.pone.0168933)
Supplement: S1 Methods — (DOCX) [file pone.0168933.s002.docx]

# Supporting Information

**Colonization in North American Arid Lands: the Journey of Agarito (*Berberis trifoliolata)* Revealed by Multilocus Molecular Data and Packrat Midden Fossil Remains**

Diego F. Angulo^1^, Leonardo D. Amarilla^2^, Ana M. Anton^2^ and Victoria Sosa^1^ ^*^

# Methods

The following section provides information of the methods used in the analysis presented in this paper.

**Sampling, DNA extraction, amplification and sequencing**

We obtained permits to collect samples for this work from the Secretaría de Medio Ambiente y Recursos Naturales, Instituto Nacional de Ecología, Dirección General de Vida Silvestre (permit number: Registro de Colección Científica VER-FLO-228-09-09).

Total genomic DNA was isolated from silica-gel-dried leaf tissue using a modified 2X CTAB method (Doyle and Doyle,1987) For DNA markers the chloroplast spacer *rpl32-trnL*^UAG^ was amplified and sequenced using the primers and protocols of Shaw *et al*. (2007). The intergenic spacer *trnH-psbA* region was amplified and sequenced using primers trnH2 (Tate and Simpson, 2003) and psbA (Sang et al, 1997) and the protocols of Shaw et al. (2005). Amplification products and DNA were purified using QIA-quick columns (Qiagen, Valencia, CA, USA) following the manufacturer’s protocols. Cleaned products were sequenced using Taq BigDye terminator cycle sequencing kits (Perkin Elmer Applied Biosystems, Foster City, CA, USA) run in an ABI 310 automated DNA sequencer (Perkin Elmer Applied Biosystems). Sequences were edited and assembled using Sequencher 4.1 (Gene Codes, Ann Arbor, MI, USA), aligned in Muscle (Edgar, 2004) and checked by eye using Se-Al v. 2.0a11 (Rambaut, 2002).

**Phylogenetic reconstruction and population genetic analyses**

As outgroups for phylogenetic reconstruction we selected *Berberis insignis* (GenBank accession GU934951), *B. chitria* (GenBank accession GU934937), and *B. umbelata* (GenBank accession GU934993), because they are considered as closely related to *B. trifoliolata* and available in GenBank database.

jModelTest v.0.1.1 (Posada, 2008) was used to identify the model of molecular evolution (TPM2uf + I) that best fit the data matrix under the Akaike Information Criterion (AIC). Four Monte Carlo Markov chains starting with a random tree were run simultaneously in two independent runs for 50 000 000 generations and sampling trees every 2 000 generations. Sample points collected prior to stationarity (convergence of likelihood scores) were eliminated as burn-in (25%). Posterior probabilities for supported clades were determined by a 50% majority-rule consensus of the retained trees.

**Divergence time**

The Markov chain Monte Carlo (MCMC) was run independently for four 50,000,000 generations, sampling every 2,000 generations. Then, TRACER 1.5 was used to assess convergence and to estimate the effective sample sizes for all parameters. Based on these results, 25% of the stored trees were discarded as burn-in, and the remaining samples were summarized as a maximum clade credibility tree in TREEANNOTATOR 1.6.1, displaying the mean divergence times and 95% highest posterior density (HPD) intervals of each age estimate (Drummond and Rambaut, 2007). These results were summarized on a single tree visualised in FIGTREE v. 1.5.4 ([http://tree.bio.ed.ac.uk/soft-ware/figtree/](http://tree.bio.ed.ac.uk/soft-%20ware/figtree/)).

**Demographic and spatial analyses**

The significance level of Tajima’s *D* , Fu’s *Fs* and *R_2_* values was calculated from 1,000 simulated samples using a coalescent algorithm (Wall and Hudson, 2001).

Bayesian skyline plot analysis was carried out using a strict molecular clock and HKY model. The MCMC procedure was run three times with 50,000,000 iterations, and the genealogy and parameters of the model were stored every 1,000 iterations. The convergence of the Beast runs was examined with Tracer version 1.6.

**AFLP analyses**

Genomic DNA was digested with *MseI* (New England BioLabs) and *Eco*RI (Promega) and ligated (T4 DNA-Ligase; Promega) to double-stranded adapters in a thermal cycler for 2 h at 37 °C. Pre-selective amplification was performed using primer pairs (*Mse*I-C and *Eco*RI-A) with a single selective nucleotide. Initially, selective primers were screened using 10 primer combinations. The six final primer combinations for the selective amplification were *Mse*I-CTG/*Eco*RI-ACA, *Mse*I-CTC/*Eco*RI-ACA, *Mse*I-CTG/*Eco*RI-AAG, *MseI*-CAT/*Eco*RI-AAG, *MseI*-CAT/*Eco*RI-AAC, and *Mse*I-CTT/*Eco*RI-ACA. Amplification products were separated on 6% polyacrylamide gels.

Bands with an identical size on the gel were assumed to be homologous. Fragments were scored as present or absent and used to construct a presence/absence matrix. We did not include AFLP fragments shorter than 50 bp because they can be highly homoplasic (Vekemans et al, 2002). Non-redundant markers were evaluated with AFLpop 1.1 software (Duchesne and Bernatchez, 2002). GenAlex 6.0 (Peakall and Smouse, 2006) was used to evaluate allele frequency and the number of markers shared among individuals with a frequency ≥5%.

The patterns of population structure (Structure analysis) were done using 10 independent runs at each K value for each hierarchical level with a burn-in period and a run length of the Monte Carlo Markov chain (MCMC), of 100,000 and 10^6^ iterations, respectively. The most likely optimal value of *K* was calculated following Evanno et al. (2005).

**Ecological Niche Modelling**

To evaluate the quality of the model, we partitioned the data into training (75%) and testing (25%) data sets. To measure the degree to which the models generated differed from what we would expect by chance and to obtain a confidence measure for the ENMs, we used the area under the receiver-operating characteristic curve (AUC) (Lobo et al. 2008).

For the past ecological niche modelling based on the fossil records of packrat middens, a database of georeferences from the "North American Packrat Midden Database" (USGS/NOAA, Version 3, <http://geochange.er.usgs.gov/midden/search.html>) was constructed with localities that included fossil records of *Berberis trifololiata*.

# References

Doyle JJ, Doyle JL (1987) A rapid DNA isolation procedure from small quantities of fresh leaf tissues. Phytochem Bull 19: 11–15.

Drummond AJ, Rambaut A (2007) BEAST: Bayesian evolutionary analysis by sampling trees. BMC Evol Biol 7: 214.

Duchesne P, Bernatchez L (2002) AFLPOP: a computer program for simulated and real population allocation based on AFLP data. Mol Ecol Notes 2: 380–383.

Edgar RC (2004) MUSCLE: multiple sequence alignment with high accuracy and high throughput. Nucleic Acids Res 32: 1792–1797.

Evanno G, Regnaut S, Goudet J (2005) Detecting the number of clusters of individuals using the software STRUCTURE: a simulation study. Mol Ecol 14: 2611–2620.

Lobo JM, Jiménez-Valverde A, Real R (2008) AUC: a misleading measure of the performance of predictive distribution models. Glob Ecol Biogeogr 17: 145–151.

Peakall R, Smouse PE (2006) GenAlEx 6: genetic analysis in Excel. Population genetic software for teaching and research. Mol Ecol Notes 6: 288–295.

Posada D (2008) jModelTest: Phylogenetic model averaging. Mol Biol Evol 25: 1253–1256.

Rambaut A (2002) Se-Al Sequence Alignment Editor, v2.0a11. Department of Zoology, University of Oxford, Oxford.

Sang T, Crawford DJ, Stuessy TF (1997) Chloroplast DNA phylogeny, reticulate evolution and biogeography of *Paeonia* (Paeoniaceae). Am J Bot 84: 1120–1136.

Shaw J, Lickey E, Beck JT, Farmer SB, Liu W, Miller J, Siripun KC, Winder CT, Schilling EE, Small RL (2005) The tortoise and the hare II: relative utility of 21 noncoding chloroplast DNA sequences for phylogenetic analysis. Am J Bot 92: 142–166.

Shaw J, Lickey EB, Schilling EE, Small RL (2007) Comparison of whole chloroplast genome sequences to choose noncoding regions for phylogenetic studies in angiosperms: the tortoise and the hare III. Am J Bot 94: 275–288.

Tate, JA, Simpson BB (2003) Paraphyly of *Tarasa* (Malvaceae) and diverse origins of the polyploid species. Syst Bot 28: 723–737.

Vekemans X, Beauwens T, Lemaire M, Roldán-Ruiz I (2002) Data from amplified fragment length polymorphism (AFLP) markers show indication of size homoplasy and of a relationship between degree of homoplasy and fragment size. Mol Ecol 11: 139–151.

Wall JD, Hudson RR (2001) Coalescent simulations and statistical tests of neutrality. Mol Biol Evol 18: 1134–1135.
