# Supplementary material for: Colonization in North American Arid Lands: The Journey of Agarito (Berberis trifoliolata) Revealed by Multilocus Molecular Data and Packrat Midden Fossil Remains
Source: PLoS One. 2017 Feb 1;12(2):e0168933. doi: 10.1371/journal.pone.0168933 (PMC5287450; doi:10.1371/journal.pone.0168933)
Supplement: S1 Table — (DOCX) [file pone.0168933.s003.docx]

**S1 Table.** List of GenBank accession for cpDNA haplotypes.

|  |  | **GenBank accesion** | | |
| --- | --- | --- | --- | --- |
| **Haplotype** | **Clones** | **rpl32-trnL** | **trnH-psbA** | |
| H1 | 17 | KY397707 | | KY397752 |
| H2 | 8 | KY397708 | | KY397762 |
| H3 | 3 | KY397687 | | KY397732 |
| H4 | 8 | KY397686 | | KY397731 |
| H5 | 1 | KY397688 | | KY397733 |
| H6 | 1 | KY397704 | | KY397749 |
| H7 | 1 | KY397709 | | KY397753 |
| H8 | 1 | KY397705 | | KY397750 |
| H9 | 1 | KY397706 | | KY397751 |
| H10 | 2 | KY397701 | | KY397746 |
| H11 | 1 | KY397700 | | KY397745 |
| H12 | 1 | KY397691 | | KY397736 |
| H13 | 27 | KY397689 | | KY397734 |
| H14 | 36 | KY397680 | | KY397725 |
| H15 | 1 | KY397717 | | KY397761 |
| H16 | 6 | KY397679 | | KY397724 |
| H17 | 1 | KY397682 | | KY397727 |
| H18 | 2 | KY397681 | | KY397726 |
| H19 | 1 | KY397683 | | KY397728 |
| H20 | 1 | KY397703 | | KY397748 |
| H21 | 5 | KY397690 | | KY397735 |
| H22 | 2 | KY397713 | | KY397757 |
| H23 | 1 | KY397712 | | KY397756 |
| H24 | 3 | KY397710 | | KY397754 |
| H25 | 1 | KY397711 | | KY397755 |
| H26 | 2 | KY397714 | | KY397758 |
| H27 | 24 | KY397695 | | KY397740 |
| H28 | 1 | KY397698 | | KY397743 |
| H29 | 1 | KY397697 | | KY397742 |
| H30 | 2 | KY397699 | | KY397744 |
| H31 | 4 | KY397715 | | KY397759 |
| H32 | 2 | KY397716 | | KY397760 |
| H33 | 1 | KY397696 | | KY397741 |
| H34 | 10 | KY397702 | | KY397747 |
| H35 | 1 | KY397694 | | KY397739 |
| H36 | 2 | KY397684 | | KY397729 |
| H37 | 12 | KY397718 | | KY397762 |
| H38 | 5 | KY397693 | | KY397738 |
| H39 | 1 | KY397692 | | KY397737 |
| H40 | 1 | KY397762 | | KY397730 |
| H41 | 1 | KY397721 | | KY397765 |
| H42 | 2 | KY397720 | | KY397764 |
| H43 | 2 | KY397719 | | KY397763 |
| H44 | 1 | KY397723 | | KY397767 |
| H45 | 1 | KY397722 | | KY397766 |
