# Supplementary material for: Colonization in North American Arid Lands: The Journey of Agarito (Berberis trifoliolata) Revealed by Multilocus Molecular Data and Packrat Midden Fossil Remains
Source: PLoS One. 2017 Feb 1;12(2):e0168933. doi: 10.1371/journal.pone.0168933 (PMC5287450; doi:10.1371/journal.pone.0168933)
Supplement: S2 Table — (DOCX) [file pone.0168933.s004.docx]

**S2 Table.** Uncorrelated environmental variables used in the current and palaeodistribution modelling in *Berberis trifoliolata*.

| Bio1 | Annual Mean Temperature |
| --- | --- |
| Bio 2 | Mean Diurnal Range (Mean of monthly (max temp - min temp)) |
| Bio 5 | Max Temperature of Warmest Month |
| Bio 6 | Min Temperature of Coldest Month |
| Bio 9 | Mean Temperature of Driest Quarter |
| Bio 12 | Annual Precipitation |
| Bio 13 | Precipitation of Wettest Month |
| Bio 14 | Precipitation of Driest Month |
| Bio 15 | Precipitation Seasonality (Coefficient of Variation) |
| Bio 18 | Precipitation of Warmest Quarter |
| Bio 19 | Precipitation of Coldest Quarter |
