# Supplementary material for: Colonization in North American Arid Lands: The Journey of Agarito (Berberis trifoliolata) Revealed by Multilocus Molecular Data and Packrat Midden Fossil Remains
Source: PLoS One. 2017 Feb 1;12(2):e0168933. doi: 10.1371/journal.pone.0168933 (PMC5287450; doi:10.1371/journal.pone.0168933)
Supplement: S3 Table — (DOCX) [file pone.0168933.s005.docx]

**S3 Table.** Samples used for Packrat Middens models prediction.

| **Sample** | **Site** | **Locality** | **Longitude (DD)** | **Latitude (DD)** | **Elevation** | **C14 age** |
| --- | --- | --- | --- | --- | --- | --- |
| BURROM1 | Burro Mesa | Big Bend National Park, Brewster Texas. USA | -103.383 | 29.267 | 1200 m | 18750 |
| DAGGER1 | Dagger Mountain | Big Bend National Park, Brewster Texas. USA | -103.1 | 29.533 | 880 m | 20000 |
| NAV3B | Navar Ranch | Hueco Mountains, El Paso Texas. USA | -106.15 | 31.9 | 1370 m | 16240 |
| SHAFT1B | Shafter | Livingston Hills, Presidio Texas. USA | -104.367 | 29.783 | 1280 m | 15670 |
| CHP3U | Streeruwitz Hills | Sierra Diablo, Hudspeth Texas. USA | -105.15 | 31.117 | 1430 m | 14290 |
